# Are specialists at risk under environmental change? Neoecological, paleoecological and phylogenetic approaches

**DOI:** 10.1111/j.1461-0248.2009.01336.x

**Published:** 2009-08

**Authors:** Audrey Colles, Lee Hsiang Liow, Andreas Prinzing

**Affiliations:** 1 Unit “Ecobio”, University Rennes 1CNRS, Campus Beaulieu, Bâtiment 14A, 35042 Rennes, France; 2 Centre for Ecological and Evolutionary Synthesis, Department of Biology, University of OsloPO BOX 1066, Blindern N-0316 Oslo, Norway

**Keywords:** Conservation biology, evolutionary dead-end, extinction and speciation, generalist-to-specialist, macroevolution, niche breadth, population decline, resource-use hypothesis, specialization-by-choice and specialization-by-constraint, species lifetime

## Abstract

The question ‘what renders a species extinction prone’ is crucial to biologists. Ecological specialization has been suggested as a major constraint impeding the response of species to environmental changes. Most neoecological studies indicate that specialists suffer declines under recent environmental changes. This was confirmed by many paleoecological studies investigating longer-term survival. However, phylogeneticists, studying the entire histories of lineages, showed that specialists are not trapped in evolutionary dead ends and could even give rise to generalists. Conclusions from these approaches diverge possibly because (i) of approach-specific biases, such as lack of standardization for sampling efforts (neoecology), lack of direct observations of specialization (paleoecology), or binary coding and prevalence of specialists (phylogenetics); (ii) neoecologists focus on habitat specialization; (iii) neoecologists focus on extinction of populations, phylogeneticists on persistence of entire clades through periods of varying extinction and speciation rates; (iv) many phylogeneticists study species in which specialization may result from a *lack* of constraints. We recommend integrating the three approaches by studying common datasets, and accounting for range-size variation among species, and we suggest novel hypotheses on why certain specialists may not be particularly at risk and consequently why certain generalists deserve no less attention from conservationists than specialists.

## Introduction

The identification of species and populations at higher extinction risk is important for developing conservation strategies in the context of current and expected environmental change. Empirical estimation of extinction risk for an individual population or species is time and resource consuming and often unfeasible; hence much research has been devoted to finding general relationships between biological traits and response to environmental change. Such relationships could then be used to predict which populations or species are at risk given environmental changes of varying magnitudes and durations.

Specialization is thought to contribute strongly to extinction risk (e.g. McKinney 1997; Biesmeijer *et al.* 2006). Our operational definition of specialization is the use of a relatively restricted subset of resources or habitats in the field by the focal species compared with other species. We use the binary categories specialization/generalization for conciseness but acknowledge that the underlying phenomena are usually continuous. We use a broad definition of extinction ranging from population declines (potentially leading to local or global extinction) to global disappearances of species or entire lineages.

The link between specialization and response to environmental change has been investigated by neoecologists studying extant species in their current environments, and by paleoecologists investigating fossil taxa in the geological past where both milder environmental fluctuations and mass extinction conditions had occurred. In a similar vein, phylogenetic biologists ask whether specialization is a dead-end leading to extinction and the hindrance of diversification.

These three approaches – neoecology, paleoecology and phylogenetic biology – frequently involve disparate temporal and spatial scales, often use inherently different data types and analytical tools, and might thus provide complementary insights into the question of whether (and why) specialists are particularly at risk under environmental change. To our best knowledge, insights from these three approaches have thus far not been synthesized. Our goal is to give a short overview of studies on the effect of specialization on the response of species to environmental change in each of these disciplines. For each discipline, we review underlying conceptual motivations, methods, data, results and limitations. We compare studies with respect to a set of criteria characterizing an ideal study on specialization and its link to decline (Table 1). We show that neoecologists, paleoecologists and phylogenetic biologists came to partly different conclusions. We discuss possible reasons for these differences, and make recommendations for future research on the link between specialization and decline.

**Table 1:** Criteria for an ideal identification of specialization, decline and for studies relating the former to the latter

*Identifying specialization*
1.Use of the environment is observed, not inferred from morphologies
2.The measure of niche breadth of species does not depend on the number of observations available for each species. This prevents abundant species from being ranked as generalists simply because they are found more frequently and, thus, in a larger number of environments. The problem does not apply to studies inferring specialization from morphological characters of species (where all individuals of a given species are usually assumed to have the same character state)
3.Information on specialization is available at the level of the species of interest. That is, specialization is not inferred from higher taxa to which that species belongs, nor from either its descendent or ancestor in a phylogenetic context
4.Niche breadth is quantified across multiple major niche axes, e.g. habitat and diet, thus approximating a true niche volume, rather than using only isolated information from single niche axes that are analysed separately. Note that this criterion is not met in any of the reviewed articles
5.Specialization is measured on a more than a binary scale; three (or preferably more) ranks are the prerequisite to identify nonlinear relationships between specialization and decline
6.An individual of a generalist species can live on a single resource or habitat type, it does not depend on multiple resource or habitat types. For instance the individual can live all its life in a forest, or it can live all its life on a meadow, it does not need to shift between forest and meadow during its existence. The criterion is obviously fulfilled for plants and parasites or phytophage larvae as they hardly move between habitats/hosts
*Identifying decline*
7.Decline measured within a given type of resource/habitat and not averaged across all those known. For instance, decline of plant species is measured only on calcareous grasslands, not for the entire region across calcareous grasslands and all other kinds of habitats. The fate of specialists and generalists is thus evaluated within the very same environmental conditions – calcareous grasslands
*Linking specialization to decline*
8.Specialization is inferred independent of decline. Either specialization is known from a period prior to the observed decline, or specialization is studied at a much larger spatial scale than decline. This reduces, but does not eliminate, the risk that the measured specialization is in itself the result of decline. Comparisons of ancestral specialization to the success of descendents were treated as cases where specialization prior to decline is known, even though the ancestral specialization is ultimately reconstructed from the descendant species. Specialization inferred from morphological characters (such as generalized/specialized mouth parts) was also treated as specialization being inferred independently of decline because such morphological characters are not likely to have changed due to decline
9.The study covers the entire range of species. As the true range is often not known or provided we used the geographic scale of the study as a proxy: the assumption is that studies at continental or global scale will usually cover entire ranges of most species included, and smaller-scale studies only rarely

We assigned binary coding to the studies we reviewed. ‘yes’ if they fulfilled the stated criterion and ‘no’ if they did not. However that finer categorization (e.g. yes, partial and no) lead to very similar results in the analysis of our literature database (Table2).

## The neoecological approach

### Why neoecologists study specialization

We define neoecology broadly as the study of ecological phenomena in the Holocene or 10 000 bp till today. We reserve the term ‘ecology’ to refer to the sum of neo- and paleoecology. Species extinctions have been common during the Holocene and are increasingly attributable to both over-hunting by humans and our drastic modifications of the environment (McKinney 1997). To ameliorate species declines, neoecologists attempt to understand how particular life-histories or interspecific interactions put species at risk of extinction under anthropogenic impact. Specialists have long been considered to be particularly susceptible to population declines; hence they have been targets of conservation efforts (Thompson 1994; Julliard *et al.* 2003).

On the neoecological time scale, species may respond to environmental change via phenotypically plastic responses, changes in the relative frequency of different phenotypes within populations or redistribution of species in space, and the tracking of habitats and climates (e.g. Ackerly 2003). We reserve the discussion of response via the establishment of new heritable traits to the The Phylogenetic Approach. Phenotypic plasticity and changes in the relative frequency of phenotypes may be relatively easy to accomplish for generalist species given their often large range of phenotypes (Spitze & Sadler 1996), while specialists might more strongly depend on spatial redistribution to track environments. Redistribution, however, is challenging for many species given the destruction of habitat corridors across landscapes; and anthropogenic large-scale transport may facilitate disperse generalists more than specialists (Prinzing *et al.* 2002a). Thus, environmental change may compel specialists to redistribute and simultaneously prevent them from doing so. Only some species already specialized on anthropogenic habitats may profit from environmental change (Munday 2004).

### Methods and data

Neoecologists usually directly observe a species’ specialization (dietary or habitat use) in its natural environment (Warren *et al.* 2001; Julliard *et al.* 2003; Koh *et al.* 2004). Inference of specialization from other traits, such as morphology, is rare (e.g. Safi & Kerth 2004 used wing morphology to infer habitat use in bats). Specialization is then compared with population trends across one to multiple decades, for which precise records on environmental changes are often available (see below). Neoecological studies cover a wide range of taxa, including insects, vertebrates, plants (see below).

### Results

#### Specialization and the risk of extinction

The correlation between habitat niche breadth, or less commonly dietary niche breadth, and the risk of extinction, is usually studied at local to regional scales [Table 2: 9(−)]. Habitat specialization is usually found to be correlated to increased extinction risk, e.g. among birds (Julliard *et al.* 2003), bats (Safi & Kerth 2004), bumblebees (Williams 2005) and plants (Walker & Preston 2006).

**Table 2:** (a) Are specialists at risk of decline? This is a tabulation of the results of our literature review (see Appendix S1) on the relationship between specialization and decline

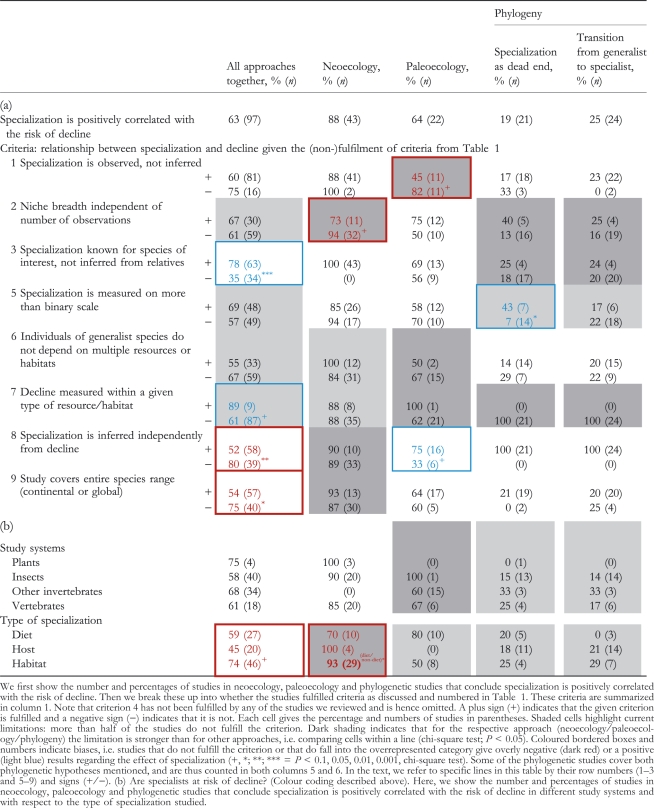

The link between diet specialization and extinction risk is less unambiguous (Table 2‘type of specialistation’). On the one hand, some studies found no relationship between dietary specialization and decline (e.g. Safi & Kerth 2004; Williams 2005 on bats and bumblebees respectively). Possibly, humans directly affect the quality and abundance of habitats, but influence dietary resources only indirectly. On the other hand, multiple studies on insect–plant interactions (phytophages, pollinators and plant-hosts) found that specialists seem to be at a higher risk of local extinction [13 of 15 cases (Appendix S1), e.g. Biesmeijer *et al.* 2006, see also Brooks & McLennan 2002]. In fact, co-extinction is common where there is mutual specialization between plants and their phytophages/pollinators (Koh *et al.* 2004; Rezende *et al.* 2007). Moving from the terrestrial to the marine realm, over-fishing is considered responsible for the majority of species disappearances and specialized predatory fish are especially at risk because of the decline of their prey species (Munday 2004).

#### Specialization and the geographic range of distribution

Specialist species exploiting narrow ecological niches are often restricted to smaller geographic ranges (Brown 1995), even though other factors such as low dispersal capability may also reduce range size (Williams *et al.* 2006). A species of narrow geographic range may face increased extinction risk under environmental change because its restricted range may only include areas where environmental conditions deteriorate for the species and not where they are neutral or improve. Butterflies of a limited geographic range, for instance, suffer three times higher risks of extinction than cosmopolitan species (Koh *et al.* 2004). Range limits such as those constrained by a minimum temperature affect specialists and generalists differently. Warren *et al.* (2001) have shown that among British butterflies, mobile-habitat generalists have expanded their range northwardly during recent decades, whereas specialists have declined. Having arrived first, the generalists may then profit from an incumbent advantage (Rosenzweig 1995) against specialists that may still arrive in the future.

Unfortunately the processes behind these geographic range patterns are not straightforward. While a large ecological niche may increase the number of regions in which a generalist species can establish, the reverse may also be true: a large range size (resulting, for instance, from efficient dispersal) exposes a specialist species to many types of resources and habitats and thus drives it to become more generalist.

#### Specialization and niche conservatism

Environmental changes can modify the distribution of species including the local disappearance of predators or superior competitors of a given species. Prey species or inferior competitors may profit by expanding into these vacated localities, possibly by increasing their repertoire of habitat types and hence become more generalist (Rosenzweig 1995). However, Losos *et al.* (1994) have shown that Caribbean lizard species did not shift to a more generalist state in the absence of a superior competitor. Similarly, Prinzing *et al.* (2002b) found that among plants poor competitors do not respond to regional shifts of stronger competitors by changing their habitat use. These results can be explained by niche conservatism, the tendency of species to retain ancestral ecological characteristics over time (Wiens & Graham 2005).

Losos *et al.* (1994) concludes that most niche changes are to be expected only when the currently used niche disappears, not when a new one becomes available. Across short, ecological time scales, niche conservatism is thought to render niche expansion difficult (Wiens & Graham 2005). Species instead respond to environmental variation by shifts in physiology or life-history (Rapoport 1982), that is, specialists do not turn into generalists (but see Fontaine *et al.* 2008). Even across macroevolutionary time scales, specialized ancestors tend to have specialist descendents (Brändle *et al.* 2002 for birds; Colles, A., Ozinga W., Hennekens, S. Schaminée, J., Bartish I., Prinzing A. unpublished data for plants, but see Sargent & Vamosi 2008 for pollinators).

### Limitations

Most studies involve populations of a given species within a single smaller region, e.g. a country [Table 2: 9(−)], and comparisons of the same species among different localities or regions in various studies are sometimes hampered by methodological differences (Julliard *et al.* 2003). Where comparisons can be made, extinction risks of populations of a species in one country may correlate only weakly to those in a neighbouring country (e.g. *R*^2^ = 0.16 for population trends of birds in France and the Netherlands; Julliard *et al.* 2003). Moreover, the species of different biogeographic origins may show different degrees of specialization (Dyer *et al.* 2007; but see Novotny *et al.* 2006). Nevertheless, the restriction of many studies to regional scales does not seem to introduce a bias as global-scale studies also tend to conclude that specialist species are declining [Table 2: 9(+)]. Note also that global scale studies may have their own drawbacks: they often rely on heterogeneous and possibly biased literature information on the distribution of species across habitats or resources.

In the majority of the studies, we do not know whether (i) individuals of generalist species actually *depend on* multiple resource or habitat types (which can be considered as an extreme form of specialization and not generalization); (ii) specialists decline *within* their preferred habitat type; or (iii) specialization may have resulted from decline. But again these do not seem to bias the observed correlation between specialization and decline (Table 2: 6, 7 and 8).

What seems more worrisome is the lack of standardization of niche breadth for sample size in many studies [Table 2: 2(−)]. If rare species are erroneously inferred to be specialists because the few observations available inevitably come from only few resource/habitat types, this may result in a pseudocorrelation between specialization and decline. Neoecological studies are largely based on insects and vertebrates, and on specialization with respect to hosts and habitats. While these taxonomic limitations did not affect the general conclusion, the focus on hosts and habitats may have lead to an overrepresentation of studies reporting a decline of specialists (Table 2: type of specialization).

Evolutionary processes are rarely accounted for in neoecological studies on declines in generalist vs. specialist species. Evolutionary responses to environmental change, including the establishment of novel, heritable phenotypes, such as those by migratory species responding to climate warming (e.g. Berthold *et al.* 1992), might take place over only a few generations. Moreover, neoecological studies might be biased towards evolutionarily derived species (species derived from the root by many phylogenetic ramifications). Existing studies primarily reflect patterns among derived species, as they make up the majority of a given extant species pool while basal species are underrepresented (Prinzing *et al.* 2004).This may introduce bias, for instance, because basal species with wide global distributions are not necessarily widely distributed within the regions they occupy, explaining the high extinction risks in these species (Prinzing *et al.* 2004,Vamosi & Wilson 2008; see also Williams *et al.* 2006).

Finally, care should be taken when habitat specialization is inferred from the number of habitat types used across largely anthropogenic landscapes, such as in much of Europe. In such landscapes, fewer habitat types are (semi-)natural, and species using these habitat types will thus likely be labelled ‘specialists’, even though in a less human-altered environment, these species may actually behave as generalists (i.e. ‘faux specialists’ sensu Brooks & McLennan 2002). A further decline of natural habitat types in anthropogenic landscapes will result in population declines in ‘specialists’, leading to an apparent relationship between specialization and decline.

### Summary

Specialists seem to suffer a triple synergistic jeopardy: a usually small initial size of local populations, an often restricted geographic range, and a limited utilization of resources and habitats (McKinney 1997). On a short, neoecological time scale, specialization appears to put species at risk under environmental change (Table 2). Nevertheless, it remains to be verified whether this pattern holds after accounting for sample size, whether it holds for dietary specialization, and whether specialists differ from generalists in their evolutionary response to environmental change. Looking back into the past, using paleoecological or phylogenetic approaches might facilitate to answer the last question.

## The paleoecological approach

### Why paleoecologists study specialization

Extinctions and originations shape biodiversity through earth’s history (Jablonski 2001). Certain traits may make some taxa more resistant to extinction; hence diversity may be a biased sample of potential taxa. In particular, Simpson’s classic ‘Rule of the survival of the relatively unspecialized’ (1944) postulates that taxa occupying wide adaptive zones are more apt to survive. Taxa which survived mass extinctions (Jablonski 2001; Kiessling & Baron-Szábo 2004) and taxa which are more resilient to extinction during ‘background’ intervals (Johnson *et al.* 1995) are thought to be non-random with respect to traits such as geographic range, niche breath, body size, complexity, among other traits (McKinney 1997). Here, we review whether specialization correlates with increased survivorship over extinction events or increased lineage longevity, emphasizing insights from publications written after McKinney’s (1997).

### Methods and data

Several authors have attempted to directly observe the relative size of the ecological niche occupied by taxa can be directly measured, such as the number of bathymetric zones or habitat types occupied (Kammer *et al.* 1998; Liow 2007a respectively), or the use of C3 and C4 plants (leaving distinct traces in the teeth of large herbivores; Sanchez *et al.* 2006; Feranec 2007). Other authors use functional morphology to infer the size of the ecological niche, such as the spectrum of food items that fossil crinoids could have consumed based the size of their filters (e.g. Baumiller 1993;Kammer *et al.* 1998). In addition, taxa which are morphologically more complex (Flessa *et al.* 1975) or those that are outliers in morphospace (Liow 2007b) had been considered specialists, although the link between general morphological features and ecological traits is not straightforward. Finally, relative specialization has been inferred from smaller geographic ranges. But because the causal relation between range size and niche breadth is not clear (see The Neoecological Approach), we consider it an untested proxy for ecological specialization and chose not to use it in our literature review.

The degree of niche specialization, observed or inferred, is then correlated with total taxon duration or taxon survivorship across previously independently identified extinction events. Taxa considered are mostly higher-order groups such as genera rather than species, although exceptions exist.

### Results

Specialization has long been thought to increase the extinction risk or decrease survivorship of a given lineage (Cope 1896; Thompson 1994). There is some evidence to substantiate this claim both over extinction events and during ‘background’ intervals, especially for feeding ecologies. For instance, omnivorous sea urchins preferentially survived over herbivorous ones across the Cretaceous-Tertiary boundary (Smith & Jeffery 1998) as did generalist insects feeding on a wider range of plant species (Labandeira *et al.* 2002). Planktonic foraminifera species which are trophic generalists (Norris 1992) and coarse-meshed and hence generalist filter-feeding crinoid genera both have longer taxon longevity (Baumiller 1993) over ‘background intervals’. Hypercarnivorous fossil canids have reduced evolutionary lifetimes (Van Valkenburgh *et al.* 2004) being more susceptible to extinction than more generalized forms (Leonard *et al.* 2007). Although correlations do not imply causation and exceptions do exist (Munoz-Duran 2002; Feranec 2007), most studies do show a tendency for diet specialization to reduce survivorship (eight of 10 studies in our literature review, Table 2: type of specialization).

Specialization has also been studied with respect to the use of habitats or other environmental units. Mammals using a greater number of biomes (Bofarull *et al.* 2008), crinoids using more habitat types as estimated using the number of facies a given taxon is preserved in (Kammer *et al.* 1998) and foraminifers able to tolerate a wider range of temperatures, salinities and nutrient settings (Keller *et al.* 1998) all have increased survivorship. However, multiple exceptions exist e.g. wider bathymetric ranges did not predict greater taxon longevities (Liow 2007a; see also Norris 1992; Jablonski & Raup 1995). In summary, four studies in our literature review suggest that species specialized on particular environments have shorter durations, while four studies did not.

Finally, rather than studying ecological specialization directly, multivariate morphology has been used to indirectly infer an average-special continuum. Results suggest that morphologically average lineages have longer evolutionary lifespans than ‘special’ or ‘outlier’ ones within clades (Liow 2007b).

### Limitations

The true taxonomic duration or survivorship of the taxa under study is difficult to estimate with precision due to the patchy nature of the fossil record. There may also be worries that certain types of specialists (e.g. living in uncommon habitats that are rarely preserved) may be seldom sampled such they appear to have shorter durations than they actually do. However, various measures are routinely used in paleobiological studies to alleviate the sampling issue, including bootstrapping (e.g. Baumiller 1993; Labandeira *et al.* 2002), confidence intervals (e.g. Labandeira *et al.* 2002), rarefaction and weighing (e.g. Liow 2007a). Also, paleontological studies estimating taxon durations (rather than comparing survivorship across extinction events) often remove extant taxa to avoid duration truncation albeit this may cause new problems: if extant species are skewed in their representation of specialists, we may be left with biased datasets.

Direct observations of resource and habitat-use are often not accessible in paleontological studies (Table 2: 1). Studies using proxies to infer specialization tend to conclude that specialist species have shorter durations more often than studies that directly observe the use of environments (Table 2: 1). Moreover, decline *within* a given resource type has been estimated in only one of the studies in our review (Labandeira *et al.* 2002). The authors of this study concluded that specialist herbivorous insects were at greater extinction risk. Where habitat/resource use has actually been observed, we often do not know whether individuals in generalist species can live on only a single one of the resource/habitat types in question or whether they require a combination of multiple types. Where we do know that they can live on a single resource, these generalist species also tend to have longer durations than specialist species (Table 2: 6). Most studies do infer specialization independently from decline, but those that do not tend to report overly weak tendency of specialists to decline [Table 2: 8(−)]. Finally, paleoecological studies largely focus on marine invertebrates and on dietary specialization, although this does not seem to bias the conclusions (Table 2).

### Summary

The majority of the paleoecological studies reviewed indicate that specialization may shorten species durations (10 of 16 studies) or reduce survivorship during major extinction events (four of six). Exceptions are more common among cases of habitat specialization. Moreover, the number of quantitative studies is relatively limited, and studies using directly observed specialization often lead to different conclusions compared with those using inferred specialization. From an evolutionary perspective, a possible response of a specialist to environmental change, other than extinction, is speciation. Alternatively, most specialists may become extinct rather than undergo speciation and most species would thus have non-specialist ancestors. These questions can be addressed by a phylogenetic approach where traits, including specialization, can be mapped onto phylogenies, and their evolution traced.

## The phylogenetic approach

### Why phylogenetic biologists study specialization

Phylogenetic trees can be used to address many macroevolutionary questions, including the direction and reversibility of evolutionary trait changes (Brooks & McLennan 2002), and for our purposes, of the trait state ‘specialist’. Specialization has long been thought to be a dead-end leading to diminished speciation and increased extinction (Cope 1896; Simpson 1944; Moran 1988). If specialization is a dead-end, this trait state should always be phylogenetically young (Futuyma & Moreno 1988). Most earlier origins of specialists should have disappeared too rapidly to leave a phylogenetic trace (Brooks & McLennan 2002). Specialists are thus expected to be relatively recent in origin and short in persistence. Specialists should unlikely be ancestors of generalists, whereas many generalists are expected to have evolved into specialists (generalists-to-specialist-hypothesis; Futuyma & Moreno 1988; Thompson 1994; Schluter 2000; Stephens & Wiens 2003).

The hypothesis of specialization as a dead-end and the generalist-to-specialist hypothesis both imply that specialists lose the capacity to adapt to novel environments due to either of two microevolutionary mechanisms (Futuyma 2001): (1) Trade-off: individuals may sacrifice their capacity to use different resources to gain competitive superiority on one of the resources/habitats. Trade-offs will drive generalists to become increasingly specialized; (2) Neutral processes: a specialist accumulates mutations that would be disadvantageous in other environments but are not selected against in the environment that the specialist actually uses. This may render future expansions of the environmental niche impossible and specialization thus becomes fixed.

### Methods and data

The reconstruction of ancestral states of specialist/generalist traits using data from extant species is a key tool used to test hypotheses on the directionality of specialist-generalist evolution. This approach is relevant because ecological specialization is to some degree heritable i.e. phylogenetically conservative (Brändle *et al.* 2002; but see Böhning-Gaese & Oberrath 1999).

The impact of environmental changes on specialists is testable when for instance specialists of a given lineage colonize a new continent and thus become exposed to a new environment (Armbruster & Baldwin 1998). However, even when no direct information on environmental changes is available, it is obvious that both biotic and abiotic environments have changed drastically throughout the lifetime of entire phylogenetic lineages, i.e. millions of years (e.g. Thompson 1994).

### Results

#### Examples of specialization as an evolutionary constraint

Cases of excessive specialization on individual host plants constraining further evolutionary diversification have been described for *Dendroctonus* wood beetles: those feeding only on a single tree genus appear only at or close to the tips of the phylogeny, in agreement with the generalist-to-specialist hypothesis (Kelley & Farrell 1998). The restriction of aphid nymphs to a single host–plant species also constrains diversification (Moran 1988). A tendency of generalists to give rise to specialists, but not the reverse, was found in walking sticks (Crespi & Sandoval 2000). Specialization is thus a handicap. Once established, specialization is rarely reversible and many ancient specialists did not survive until present.

#### Counterexamples

However, numerous studies fail to confirm the generalist-to-specialist and the dead-end hypotheses. For instance, in the seed beetle genus *Stator*, species specialized on *Acacia* may evolve into generalists, but also into specialists using a novel host plant (Morse & Farrell 2005). Similarly, butterflies of the tribe Nymphalini were originally specialists, but later evolved into generalists or specialized on novel hosts (Janz *et al.* 2001). Hence, it was concluded that ‘specialization is not a path of no return’ (Janz *et al.* 2001). Plant-pollinator relationships may be exemplified by the continental-African euphorbia *Dalechampia*, pollinated by specialized pollinators collecting the plant’s resin. After migration to Madagascar, *Dalechampia* interacted with a more diverse and generalist pollinator fauna as specialist pollinators were not available (Armbruster & Baldwin 1998). Here, a specialist plant responded to its novel environment by becoming more generalist and perhaps simultaneously escaping its previous natural enemies. Nevertheless, Rezende *et al.* (2007) found within a local pollinator community that specialized mutualistic interactions were phylogenetically conserved, i.e. specialist ancestors neither evolved into generalist descendants nor did they go extinct. Such phylogenetic patterns within communities may reflect larger-scale evolutionary processes of entire lineages (Prinzing *et al.* 2008).

Overall, some 80% of the studies reviewed indicate that specialists are well capable of changing back to generalists, and where they did not, they nevertheless survived, contradicting the generalist-to-specialist hypothesis and the dead-end hypothesis respectively (Table 2; column ‘phylogeny’). Earlier reviews (Futuyma & Moreno 1988; Thompson 1994; Schluter 2000) have already pointed this direction. Based on our more extensive review we are able to explore whether this lack of evidence for specialization as an extinction risk may result from particular limitations or biases (Why the Three Approaches Come to Different Conclusions: From Biases to Mechanisms).

### Limitations

Limitations of the phylogenetic approach stem mainly from uncertainty and biases in reconstructing ancestral states. Stireman (2005) showed that standard methods like parsimony or symmetric maximum likelihood tend to assign specialist states to ancestors and generalist states to descendants. This bias may be caused by unequal numbers of specialist and generalist species in data, where most reconstructed trait transitions go from the rarer to the more common trait state (Nosil & Mooers 2005). We found evidence for a bias due to the relative frequency of specialists and non-specialists, but in the opposite direction: all studies in which specialists are more numerous than generalists concluded that specialization is not a dead-end and specialists can already be found among ancestors (Appendix S1) while 33% of the remaining studies concluded specialization to be a dead-end (*n*= 15, χ^2^ = 3.46, d.f. = 1, *P*= 0.063). The accuracy of character-state reconstructions have been studied by comparing true character states observed in the fossil record or *in vitro* evolution of microbe lineages to reconstructed states: One study confirmed the correctness of the reconstructions (Polly 2001) but two others did not, in particular where there was a strong evolutionary trend of traits across clades (Oakley & Cunningham 2000; Webster & Purvis 2002). Character state reconstructions as weighted averages within-lineage across descendants of a given ancestor appear to be more robust (Oakley & Cunningham 2000 and references therein) but do not permit testing of the polarity of trait changes. They do however permit testing for specialization as a dead-end (i.e. specialist ancestors having fewer descendants than their generalist sister-taxa). Where this has been performed (*n*= 5), the results were not drastically different from those in studies applying character-state reconstructions across entire lineages (specialization identified as dead end in 40% vs. 13% of the studies). Studies, in which specialization of at least some of the ancestral species could be directly observed have similar results to those where specialization was reconstructed (Table 2: 1).

Results also may depend on whether specialization/generalization is coded by discrete or continuous variables: emydid turtles ancestors are assigned as aquatic, i.e. habitat specialist using discrete character coding, but are reconstructed as semi-terrestrial, i.e. generalist, using continuous coding (Stephens & Wiens 2003– but note that treating semi-terrestrials as generalists may not fit our criterion 6 in Table 1). Discrete variables hinder the reconstruction of ‘intermediate’ values at ancestral nodes, and the number of changes of the trait may be underestimated. Most authors use discrete variables to characterize specialization (which is naturally a continuous phenomenon), resulting in a bias in favour of increased reversibility of specialization (Stephens & Wiens 2003). This is confirmed by our review: binary definition of specialization corresponds to an overly low tendency of specialists to decline (Table 2: 5).

Many phylogenetic studies lack the standardization of niche breadth measures for sample size, but this deficiency does not change the assessment of consequences of specialization (Table 2: 2). However, none of the reviewed studies analysed the success of specialists within only a given resource or habitat type (Table 2: 7), hence we do not know whether specialization leads to dead-ends more often under such scenarios.

Finally, phylogenetic studies on specialization are largely limited to insects using plant or animal hosts, i.e. a specialization on another taxon (Table 2). Hosts may induce changes in the phytophage or parasite physiology, rendering host shift increasingly difficult, and specialist phytophages or parasites may indeed find themselves in a dead end. On the other hand, hosts may diversify, and specialist phytophages or parasites may profit from an incumbent advantage and diversify along with their hosts. Such advantages and disadvantages from host-specialization may compensate for each other, and in fact studies on host specialization do not find that specialists decline more often than studies on other types of specialization (Table 2). Studies of host specialization often measure host ranges at different taxonomic scales, e.g. from tribes within a plant genus to all Angiosperms. However, we found that the results of studies did not depend on the taxonomic level of the host specified: specialists were regardless rarely at risk.

### Summary

Specialization does occasionally limit the capacity of a species to persist across environmental changes, but many studies also question the idea that specialization is an evolutionary dead-end. The different conclusions do not merely reflect differences in the taxonomic groups studied (Table 2). In fact, two studies on phytophagous beetles have opposing results (Kelley & Farrel 1998; Morse & Farrell 2005). That specialization may not be evolutionarily disadvantageous seems surprising given the costs of specialization. However, the benefits of increased resource use efficiency may outweigh the costs of increased resource specialization, even over long periods (Futuyma 2001). For instance, environmental change leading to the disappearance of one host–plant species may simultaneously permit the appearance of another plant species equally exploitable for the given specialist phytophage (Brooks & McLennan 2002). These specialists, sometimes termed ‘faux generalists’ specialize on particular biochemical and morphological plant phenotypes rather than strictly on individual plant species. The evolutionary constraint due to specialization needs to be verified for each study system separately, while accounting for possible methodological biases in ancestral character-state reconstruction. Nevertheless it seems safe to conclude that, from a phylogenetic perspective, specialization does not, in general, put a species at greater risk in a changing world.

## Why the three approaches come to different conclusions: from biases to mechanisms

Specialization appears detrimental for the persistence of species under current environmental change. Many paleoecological studies point to similar conclusions although species level studies are rare and proxies for specialization may not accurately reflect realized ecological specialization. On the other hand, most phylogenetic studies show that species do not suffer from being specialists; they can avoid extinction and adapt, for instance they respond to the challenges of a new biotic environment when colonizing a new continent. To the best of our knowledge, neoecologists were not aware of these phylogenetic observations when discussing the conservation needs of specialists.

Why do three different approaches, studying the same phenomenon, come to different conclusions, and how could they be reconciled to come to pertinent predictions on the future of specialists and generalists under anthropogenic change? We will start with biases in data and methods, and then move on explanations invoking different mechanisms.

### Different biases?

In neoecological studies, niche breadth measures are seldom standardized such that the tendency of specialists to decline is probably over-estimated. Because neoecological studies are often restricted to local or regional scales, specialists may appear to be more often on the decline than they may be if global data were available (analysis across approaches, Table 2: 9). Paleoecological studies may also over-estimate the tendency of specialists to decline due to the indirect inference of specialization of species from proxies such as morphology (analyses within paleoecology, Table 2: 1). Finally, phylogenetic approaches possibly underestimate the tendency of specialists to decline due to the binary categorization of specialization (analysis within phylogeny, Table 2: 5), the inference of character states of ancestral species from descendents, the lack of information on decline within habitat or resource types (analysis across approaches, Table 2: 1 and 7), or the numerical dominance of specialist species (potentially biasing the reconstruction of ancestral states, see above).

Taking into account only the (few) studies that do not suffer from the listed biases renders the effects of specialization more similar across different approaches. But proportions are still different: specialists decline according to 73% of the unbiased neoecological approaches, but only according 45% and 43% of the unbiased paleoecological and phylogenetic approaches respectively.

### Different study systems?

The three approaches often considered very different taxa. However, our analysis showed that this does not bias the results: conclusions are overall consistent across different taxa (Table 2; but note low sample sizes in some taxa). For instance, paleoecological studies largely focus on marine invertebrates because of their higher preservation rates and neoecological studies often focus on insects or vertebrates, but the two approaches reach similar conclusions nevertheless (Table 2). The type of specialization, in contrast, may be a major contributor to different conclusions among approaches. Habitat specialists found to be more at risk than dietary specialists in neoecological studies (Table 2). Dietary specialists may be specialized on distinct traits of their food-species (e.g. their prey or host) rather than on the food-species *per se*. Hence the disappearance of a food species may be more easily compensated by the usage of newly appearing species with the same traits (‘faux generalists’, see Brooks & McLennan 2002). Habitats, in contrast, may be more complex than food items, and disappearance of one habitat type may be more rarely compensated by appearance of another. However, habitat specialization is not always correlated with extinction risk in paleontological datasets.

Differences in study systems among the three approaches seem to explain in part why they come to different conclusions on the relationship between specialization and decline. However, even for the same study systems neoecological studies tend to indicate strongest declines in specialists, phylogenetic studies weakest declines (Table 2; but note lower sample sizes). Differences between results from neoecological, paleoecological and phylogenetic studies might thus also be due to different temporal and spatial scales of the studies and in the processes responsible for specialization, generalization and extinction.

### Different temporal scales and amplitudes of environmental change?

Neoecologists typically study environmental impacts on a short-time scale (10^2^ years), aiming to identify species facing risk of extinction under current anthropogenic impacts. Many paleoecologists are concerned with survivorship of taxa on a geological time scale (*c*. 10^5^–10^8^ years) over both long background time intervals but also shorter periods of mass extinctions. Finally, phylogenetic biologists study diversification throughout the lifetime of entire lineages (*c*. 10^6^–10^8^ years). Neoecological and some paleoecological studies often focus on catastrophic or severe environmental changes, while phylogenetic studies tend to integrate over the entire life time of species. However, as outlined above, many paleoecological studies, studying long-term background extinctions between major catastrophes still concluded that specialists are at greater extinction risk than generalists, suggesting that differences in temporal scale in themselves do not confound results.

### Different causes of specialization or of generalization?

Some species are specialized due to physiological or morphological constraints that prevent them from being more generalist (for instance dietary specialization in phytophages, Bernays 1998). However, behavioural ecologists have long recognized that individuals make decisions throughout their lives: an individual capable of using many resource types may often *decide* to specialize on one type of resource (Ward 1986; Prinzing 2003). Generalists may use suboptimal resources because they cannot afford to wait till they find their preferred, optimal resource type (Ward 1986). Such individual decision making processes may also drive species-level specialization: species with life-histories restricting search time cannot be choosy and are hence generalists (Prinzing 2003; note that decision making is not restricted to animals – plants do so too, for instance seeds germinate or not as a function of changes in ambient temperature or day length; Karban 2008). A specialist species may thus not have *more* physiological or morphological constraints, but *fewer* search-time constraints, such that its individuals have the *option* of specializing on the optimal resource or habitat type. They thus have the *choice* of becoming more generalist or switching to specialization on another resource. Hence, specialization would be much less of a burden in a changing environment.

Specialization due to lack of search-time constraints has been considered to be particularly important in host-specialization (Ward 1986). This might explain why many phylogenetic biologists, often studying host specialization, have found that specialization does not put species at risk. In contrast, paleoecologists have often inferred specialization from morphological characters imposing a constraint on resource use, especially food items, and often found specialists to be at risk. Understanding whether a given specialist is specialized due to the lack of search-time constraints or due to physiological or morphological constraints on resource use is thus crucial.

Generalization may have different causes, too. A species may be a generalist either because its individuals are generalist or because different individuals are specialized on different resource or habitat types (e.g. Ben-Halima *et al.* 1985). In the latter case; the specialized individuals would profit from all advantages of being specialized (such as efficiency of resource use), and the species as a whole can nevertheless shift between different resources if one declines.

While we know of no study on correlates of species decline that has separated individual-level from species-level niche breadth, it is obvious that species-level generalization as the sum of individual-level specializations cannot be detected if a single mean value serves to characterize an entire species. Interestingly, our analyses show that studies inferring generalization of species from mean morphology [i.e. all studies classified under criterion 1(−)] nevertheless rank generalist species as equally or more successful than specialist species (Table 2; across-approach analyses, within paleocology analyses) – despite the fact that these generalist species are assumed to have generalist individuals. Also, species-level generalization as the sum of individual-level specializations is more likely in species where each individual encounters only a single-host patch (Futuyma 2001), e.g. in plants or parasites or in phytophages on hosts. However, our analysis provides no evidence that generalist species in these groups are more successful than specialists compared with other groups (Table 2).

### Different causes of extinction?

We broadly defined extinction risk to mean population declines and/or global disappearances of species or lineages. However, the reduced evolutionary lifespans of specialist *species* under major environmental change do not necessarily imply the extinction of entire specialist *lineages*. Vrba (1987) proposed the resource-use hypothesis, stating that specialist taxa exploiting a narrow range of resources will go through periods of population decline and subdivision under environmental change, triggering speciation by reproductive isolation. Paleoecological and population biological observations seem to lend some evidence to this (Kammer *et al.* 1998; Zayed *et al.* 2005). Generalist taxa, in contrast, seldom undergo such drastic reductions in population sizes, that is, conditions that may spur speciation. This explanation critically depends on the assumption that specialists remain specialists and generalists remain generalists under environmental change, which may not necessarily be true (cf. our above discussion of causes of specialization). Nevertheless, in accordance with this explanation, large mammals with relatively specialized diets have higher speciation rates (Vrba 1987). Overall, even though specialist species may be at greater risk under environmental change than generalists, the increased extinction rates in specialist clades may be offset by increased speciation rates.

## Recommendations

Our review has revealed multiple open questions, some of more technical nature, others directing to entirely new mechanistic hypotheses to be tested in future. We recommend the following for future work to make the study of specialization vs. decline a more interdisciplinary one with results that are more general.

### Directly quantify specialization in an unbiased way

We suggest that specialization should be directly observed instead of being inferred – wherever possible (Table 1; criterion 1). The measure of niche breadth should be independent of the size of the sample (criterion 2). We found analyses where these two criteria are not fulfilled are often biased. In addition, we recommend aiming at characterizing habitat specialization based on environmental factors which exist in human-made habitats just as much as in more natural habitats. This reduces the risk of misclassifying generalists that use habitat types absent from modern anthropogenic landscapes as specialists. For plants, for instance, light, temperature, soil moisture, soil pH, soil fertility, salt concentrations and grazing have been shown to efficiently predict species distribution and assessments of preferences of species along these gradients are available e.g. for central Europe (Ellenberg *et al.* 1992). These data represent only niche positions, but the niche breadth can then be inferred from the abundant data on composition of local communities: species that co-occur only with other species of the same niche position are likely highly specialized. (Ellenberg *et al.* 1992; Synbiosys, http://www.synbiosys.alterra.nl). For organisms for which such databases do not exist, climate envelopes, phenological breadth or ecomorphological criteria may be suitable assessments of the geographical, local or fundamental niche respectively (Safi & Kerth 2004; Jiguet *et al.* 2007).

### Use common datasets to integrate the three approaches and to circumvent approach-specific biases

Applying phylogenetic approaches to neoecological or paleobiological datasets, particularly those involving species with living representatives or relatives, can facilitate illuminate some issues. Neocological and paleobiogical datasets often cover more lineages than normally used by most phylogeneticists (for instance all birds or all angiosperms in a region; e.g. Klotz *et al.* 2002). In using these datasets, less phylogenetic information, such as branch lengths, may be readily available, but sample sizes are high, and analyses such as sister-clade comparisons could lead to robust results (Oakley & Cunningham 2000; see Mitter *et al.* 1988 for an example of linking resource use to diversification across sister clades). Neoecologists, on the other hand, may begin to understand how the present-day relationship between specialization and extinction risk depends on the evolutionary history of species. Cross-disciplinary studies have already given us new insights, for instance when comparing basal and derived species within lineages (The neoecological approach-Limitations). Another example are tropical phytophages which may be more specialized across plant lineages (Dyer *et al.* 2007, but see Novotny *et al.* 2006), which, if confirmed, might be an additional explanation of extinction risks faced by tropical species. Finally, paleoecological studies can likewise benefit from using phylogenetic information for clarifying the statistical non-independence of related taxa and from incorporating shorter time-scale mechanisms illuminated by neoecological studies.

### Account for range-size variation among species

Analyses should account for the confounding effect of geographic range. Although broadly ranging species have a potentially large global niche breadth which possibly decreases extinction risk, simply having a large range also reduces extinction risk. After accounting for range size, the effect of ecological specialization on taxon longevity may disappear (Liow 2007a). Besides these confounding effects, local risks and global risks may also be different in nature and hence studies can benefit from encompassing as many spatial scales as possible.

### Understand why some specialists may not be at risk

We suggest two major hypotheses why specialists may not be at risk. The first is that specialization on specific resources due to physiological or morphological constraints puts species at risk compared with generalists, while specialization due to lack of search-time constraints does not (because it is reversible, see above). Both types of specialization might be readily identifiable. For instance, organisms lacking protective structures (such as cuticles) and tolerance mechanisms might be specialized due to physical constraints, while organisms with slow life-histories may be specialized due to lack of search-time limitations (Prinzing 2003).

The second hypothesis is that speciation may compensate for extinction in specialist lineages. Some higher-level taxa are not observed to suffer declines in paleoecological studies perhaps because speciation occurred over environmental changes. Recent methodological advances in separating the phylogenetic fingerprints of extinction and speciation (e.g. Maddison *et al.* 2007 and references therein) may facilitate to test this scenario, but to our knowledge, have not been applied to studies involving specialization and decline. Encouragingly, even neoecologists may approach these potentially pertinent evolutionary mechanisms by studying the first steps of speciation, e.g. in terms of subspecies formation.

Both hypotheses have implications for evolutionary ecology. The first suggests that for some species specialization is not costly. The costs of being a generalist, such as inefficient resource use, may thus be much more important than previously thought. The second suggests that the species level may sometimes not be the appropriate level of analysis. Specialization may persist throughout evolution not because it facilitates the survival of species having specializations but because it promotes speciation.

The above recommendations will permit identification of situations where specialists are indeed at risk: conservation efforts should be focalized on the particular resources and habitats of these specialists. Moreover, the final recommendation will establish a bridge between conservation biologists studying correlates of being at risk and evolutionary biologists studying costs and benefits of species niche use.

## SUPPORTING INFORMATION

**Appendix S1** References of articles analyzed in table 2 and full data matrix.
